# Prognostic Factors for Cervical Cancer in Asian Populations: A Scoping Review of Research From 2013 to 2023

**DOI:** 10.7759/cureus.71359

**Published:** 2024-10-13

**Authors:** Syed S Abrar, Seoparjoo Azmel Mohd Isa, Suhaily Mohd Hairon, Najib M Yaacob, Mohd Pazudin Ismail

**Affiliations:** 1 Department of Community Medicine, School of Medical Sciences, Universiti Sains Malaysia, Kota Bharu, MYS; 2 Department of Pathology, School of Medical Sciences, Universiti Sains Malaysia, Kota Bharu, MYS; 3 Department of Biostatistics and Research Methodology, School of Medical Sciences, Universiti Sains Malaysia, Kota Bharu, MYS; 4 Department of Gynecology and Obstetrics, School of Medical Sciences, Universiti Sains Malaysia, Kota Bharu, MYS

**Keywords:** asia, cervical, cervical cancer, prognosis, prognostic factor

## Abstract

Cervical cancer is the fourth most common cancer among women worldwide, with particularly high incidence and mortality rates in low- and middle-income countries, with Asia reporting the highest number of cases in 2022. Despite this significant burden, the prognostic factors specific to Asian populations remain underexplored. This scoping review aimed to identify and evaluate prognostic factors associated with cervical cancer outcomes in Asia, focusing on clinical, socio-demographic, and treatment-related variables. The review followed the Arksey and O'Malley framework and included 44 studies published between 2013 and 2023. The majority of research was concentrated in East Asia, particularly in China, Japan, and South Korea. Key prognostic factors affecting overall survival and disease-free survival included tumor size, histology, age, lymphovascular invasion, and lymph node metastasis. Non-squamous cell carcinoma histology, especially adenocarcinoma, was consistently linked to poorer outcomes. Older age and medical comorbidities, such as anemia and diabetes, also negatively impacted survival. Treatment-related factors, though less frequently reported, demonstrated the significance of adjuvant therapy, chemotherapy, and treatment intensity in improving outcomes. This review underscores the complexity of cervical cancer prognosis in Asian populations and highlights the need for targeted research and region-specific interventions to address the rising incidence of cervical cancer. It also highlights the scarcity of research on cervical cancer prognostic factors in West, Central, and South Asian countries. Future research should aim to address the gaps in understanding treatment-related factors and explore the potential for region-specific interventions to improve outcomes in cervical cancer across Asia.

## Introduction and background

Cervical cancer is the fourth most common cancer among women worldwide, with particularly high incidence and mortality rates in low- and middle-income countries, including Sub-Saharan Africa, Central America, and Southeast Asia [1]. In 2022, there were approximately 660,000 new cases and nearly 350,000 deaths, with 94% of these deaths occurring in low- and middle-income countries [1]. Asia reported the highest number of cervical cancer cases in 2022, accounting for 60% of the global total, and also recorded over half of the deaths, followed by Africa and Latin America [2].

In Asia, cervical cancer is the fourth most common cancer among women and the third most prevalent among those aged 15 to 44. Southern Asia has the highest number of cases, with the highest age-standardized incidence rates observed in the Maldives, Indonesia, and Myanmar, among others. The highest age-standardized mortality rates are found in Indonesia, Myanmar, the Maldives, Mongolia, and India, with Southeast Asia exhibiting the highest mortality rates in Indonesia and Myanmar [3].

Prognostic factors linked to cervical cancer survival include clinical characteristics, treatment-related factors, and sociodemographic variables. Commonly observed prognostic factors are age, International Federation of Gynecology and Obstetrics (FIGO) stage, histology, tumor size, and lymph node status [4-10]. For instance, age has been identified as an important prognostic factor in several studies. A German study evaluating 15,685 patients found improved survival rates in younger age groups [9]. An analysis of 24,562 patients from the Surveillance, Epidemiology, and End Results (SEER) database observed worse survival outcomes in patients with adenocarcinoma at both early and advanced stages [10].

Given the rising incidence of cervical cancer in Asian countries, there is a need to better understand the factors affecting prognosis in these populations. This review aims to explore the existing literature and identify both common and novel prognostic factors associated with cervical cancer in Asian countries.

## Review

Search strategy and selection criteria

This scoping review aimed to identify prognostic factors for cervical cancer in Asian countries. The review followed the Arksey and O'Malley framework, comprising five systematic steps: developing a broad research question; comprehensively identifying relevant studies; selecting studies that meet predefined inclusion and exclusion criteria; extracting data; and synthesizing and reporting the results [11].

The research question, "What are the prognostic factors for cervical cancer in Asian countries?" was developed using the Population/Concept/Context (PCC) strategy. Relevant studies were identified through a comprehensive search in PubMed, using search terms such as “prognostic factors” OR “prognosis” AND “cervical cancer” OR “cervical carcinoma” AND “Asia.” The search was limited to articles published between 2013 and 2023 to ensure the collection of recent data. Only studies published in the English language were considered.

Screening

There were two phases in the selection procedure. SSA and SAI, the two authors involved in the screening process, independently reviewed the titles and abstracts of all articles retrieved from the PubMed search. Each author applied the pre-established inclusion and exclusion criteria. Articles were included in the initial screening if they addressed prognostic variables for cervical cancer in Asian nations. Editorials, letters, conference abstracts, news pieces, and reviews were excluded, as well as abstracts deemed irrelevant to the research question.

After the initial screening, both authors compared their selections. Any discrepancies between their choices were discussed and resolved through consensus. Articles that both authors deemed relevant were then included for full-text review. In this second phase, full articles were reviewed to ensure they met all inclusion criteria and addressed the specific research question. Only studies fitting the research question were included in the final analysis. 

Data extraction and management

Microsoft Excel (Microsoft Corporation, Redmond, Washington, United States) was used for data management, and the findings from the included research were exported for further analysis. The extracted data included general study details (e.g., authors, publication date, location), sample population, sample size, and identified prognostic variables. Only outcomes that met the statistically significant threshold of P < 0.05 and were significant factors in multivariate analysis were considered. Consistency in data extraction was achieved by comparing the data obtained by the two researchers, with any discrepancies being discussed and resolved.

Results

**Figure 1:**
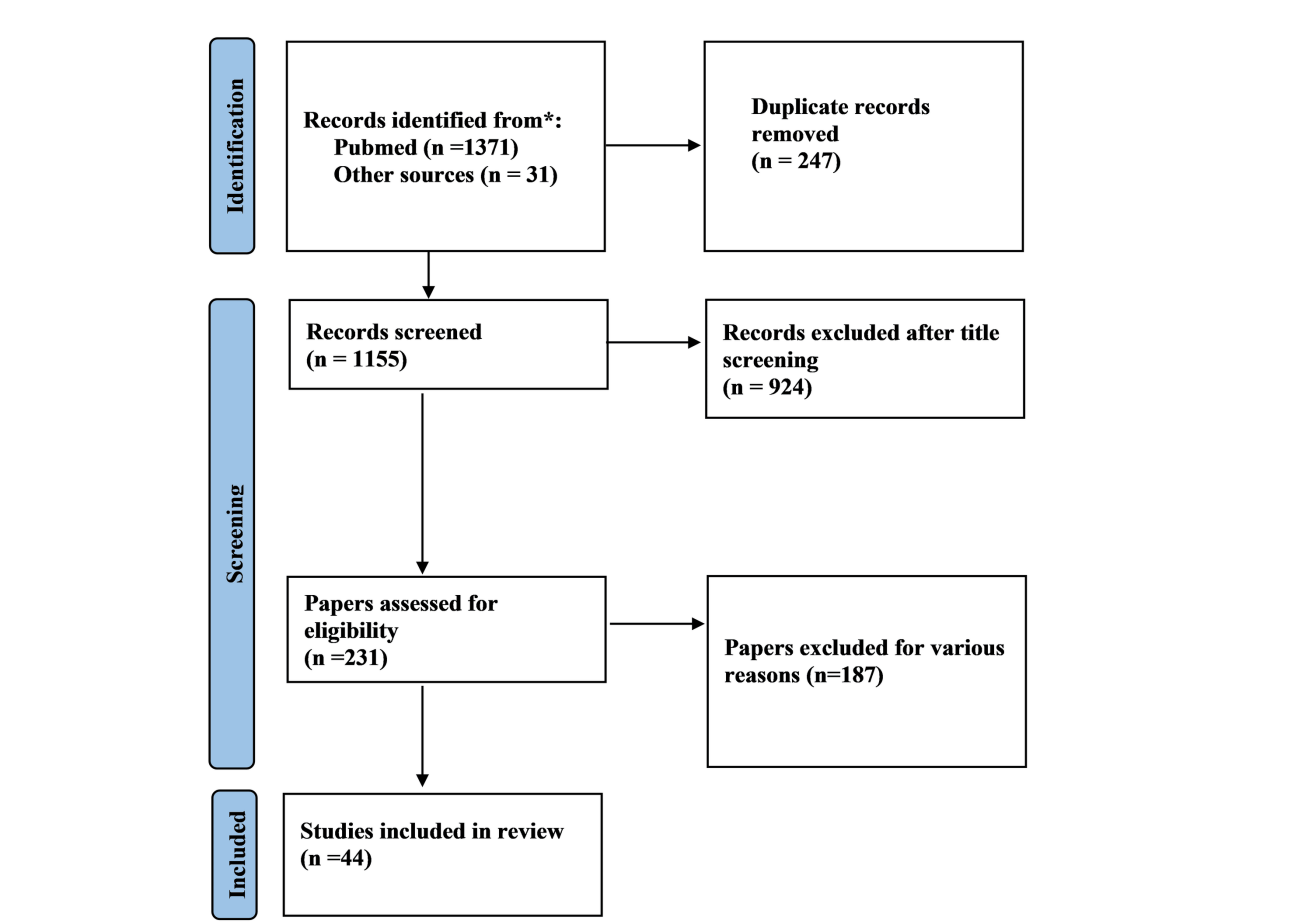
Flow diagram depicting the study selection process

The initial search yielded 1,371 papers, with an additional 31 papers retrieved from other sources. After removing duplicates, 1,155 articles were available for further screening. Title screening identified 231 papers eligible for abstract review. Ultimately, 44 articles were included in the final review. A flowchart based on the Preferred Reporting Items for Systematic Reviews and Meta-Analyses (PRISMA), illustrating the screening and selection process, is shown below (Figure 1).

General Description of Selected Studies

Of the 44 studies included in this scoping review, 28 were single-institutional, 13 were multi-institutional, and three were based on nationwide data. A description of all the studies included in the review is listed in Table 1.

**Table 1:** Description of studies included in the review AC: adenocarcinoma; CSS: cancer-specific survival; DFS: disease-free survival; DMFS: distant metastases-free survival; DSI: deep stromal invasion; FIGO: International Federation of Gynecology and Obstetrics; HPV: human papillomavirus; LFFS: local failure-free survival; LN: lymph node: LNM: lymph node metastasis; LVI: lymphovascular invasion; LVSI: lymphovascular space invasion; nSUVmax: nodal maximum standardized uptake value; OS: overall survival; OTT: overall treatment time; PFS: progression-free survival; pSUVmax: primary tumor maximum standardized uptake value; RFS: recurrence-free survival; SCC: squamous cell carcinoma; PI: parametrial involvement; mPLN: metastatic pelvic LN

Reference	Country	Study design	Sample size	Data source	Study population	Key findings
Lee et al. (2013) [12]	South Korea	Retrospective cohort	1,702	Multi-institutional	Early-stage cervical cancer patients	Age, number of positive pelvic LNs, parametrial invasion, and LVI.
Shim et al. (2013) [13]	South Korea	Retrospective cohort	209	Single institution	Locally advanced cervical cancer patients	OS: Histology, tumor size, and para-aortic LNM (as determined by MRI) were independent predictors, while PET/CT prior to CCRT was not.
Tsubamoto et al. (2013) [14]	Japan	Retrospective cohort	73	Single institution	Locally advanced cervical cancer patients	Tumor size, pathological response, and LNM.
Intaraphet et al. (2014) [15]	Thailand	Retrospective cohort	130	Single institution	Patients with small cell neuroendocrine carcinoma of the uterine cervix	Extensive stromal invasion and age over 60 at diagnosis were associated with lower survival rates in patients with early-stage disease. Age at diagnosis and FIGO stage IV were associated with worse survival rates among individuals with advanced-stage disease.
Wu et al. (2014) [16]	Taiwan	Retrospective cohort	55	Single institution	Cervical cancer patients	OS: Age < 53 years, FIGO stage I–II, and treatment with extended-field radiotherapy (EFRT) were independent predictors.
Ariga et al. (2015) [17]	Japan	Retrospective cohort	667	Multi-institutional	Early-stage cervical cancer patients	OS and DFS: Tumor diameter and nodal status were significant prognostic indicators.
Chen et al. (2015) [18]	Taiwan	Retrospective cohort	125	Single institution	Locally advanced cervical cancer patients	OS: AC, initial LNM, and weekly cisplatin doses. DFS: AC, LFFS: AC, weekly cisplatin doses, and higher pretreatment hemoglobin levels.
Fujiwara et al. (2015) [19]	Japan	Retrospective cohort	178	Single institution	FIGO IB2–IVA cervical cancer patients	PFS: Histologic type, maximum tumor diameter, and pretreatment hemoglobin level.
Kuo et al. (2015) [20]	Taiwan	Retrospective cohort	2,946	Nationwide Taiwan Cancer Registry Database	Early-stage cervical cancer patients	OS and CSS: Diabetes mellitus is an independent unfavorable prognostic factor.
Lee et al. (2015) [21]	South Korea	Retrospective cohort	80,766	National Database	Cervical cancer patients	AC was an independent negative prognostic factor for survival.
Li et al. (2015) [22]	China	Retrospective cohort	30	Multi-institutional	Patients with small cell carcinoma of the cervix	DFS and OS: Advanced FIGO stage was the only independent risk factor.
Okuma et al. (2016) [23]	Japan	Retrospective cohort	71	Single institution	Cervical cancer patients	DFS was significantly associated with HPV (detected vs. not detected). Patients in whom HPV was not detected had the worst prognosis.
Shen et al. (2016) [24]	China	Retrospective cohort	112	Single institution	Patients with early-stage cervical SCC	OS: High-level CPE expression.
Twu et al. (2016) [25]	Taiwan	Retrospective cohort	1,132	Multi-institutional	Patients with early-stage cervical AC and adenosquamous carcinoma	RFS: stage II, DSI, LVSI, positive pelvic (LN), and parametrial involvement (PI). CSS: DSI, PI, and positive PLN.
Usami et al. (2016) [26]	Japan	Retrospective cohort	111	Single institution	Late-stage cervical cancer patients	Prognostic factors: The performance status, site of metastases (only lymph node or other metastases), and local stage.
Han et al. (2017) [27]	China	Retrospective cohort	136	Single institution	Patients with cervical AC.	Pelvic LN involvement and vaginal invasion are independent adverse risk factors for survival.
He et al. (2017) [28]	China	Retrospective cohort	1,863	Multi-institutional	Early-stage cervical cancer patients	LVSI, deep stromal invasion, and LN metastasis were independent prognostic factors.
Jung et al. (2017) [29]	South Korea	Retrospective cohort	1,113	Single institution	Cervical cancer patients	DFS: AC was a significant independent factor.
Lin et al. (2017) [30]	Taiwan	Retrospective cohort	2,594	Nationwide	Cervical cancer patients	CSS and OS: Old age, non-SCC type, high-grade histology, increased tumor size, advanced FIGO. stage, and prolonged OTT.
Yanaranop et al. (2017) [31]	Thailand	Retrospective cohort	316	Single institution	Early-stage cervical cancer patients	RFS: Cervical stroma invasion (CSI) and LVSI were the significant prognostic factors. OS: CSI was the only significant prognostic factor.
Zheng et al. (2017) [32]	China	Retrospective cohort	795	Single institution	Cervical cancer patients	Prognostic factor: Body mass index, albumin, platelet, leukocyte, tumor differentiation, and the status of the pelvic LN.
Feng et al. (2018) [33]	China	Retrospective cohort	82	Single institution	Patients with neuroendocrine carcinoma of the cervix.	LNM and FIGO stages were independent prognostic factors.
Kwon et al. (2018) [34]	South Korea	Retrospective cohort	259	Multi-institutional	Early-stage cervical cancer patients	DFS and DMFS: mPLN (metastatic pelvic LN), LVI, and non-SCC were unfavorable.
Liu et al. (2018) [35]	China	Retrospective cohort	98	Single institution	Patients with stage I–III cervical carcinoma	OS: Anemia before radiotherapy and tumor size.
Shimamoto et al. (2018) [36]	Japan	Retrospective cohort	959	Single institution	Cervical cancer patients	Prognostic factors: Clinical stage, histological type, treatment intensity, and primary surgery.
Cao et al. (2019) [37]	China	Retrospective cohort	5,181	Single institution	Early-stage cervical cancer patients	For RFS: AC patients had a 2.6 times higher risk compared to SCC. Adenosquamous carcinoma patients had a 2.1 times higher risk compared to SCC. For OS: AC patients had a 3 times higher risk of death compared to SCC. Adenosquamous carcinoma patients had a 2.3 times higher risk of death compared to SCC.
Matsuo et al. (2019) [38]	Japan	Retrospective cohort	5,964	Multi-institutional	Early-stage cervical cancer patients	Hospital volume for radical hysterectomy may be a prognostic factor. Surgery at high-volume centers is associated with improved survival.
Nuranna et al. (2019) [39]	Indonesia	Retrospective cohort	1,303	Single institution	Cervical cancer patients	Cox regression analysis showed that the factor affecting survival was the cancer stage.
Wang et al. (2019) [40]	China	Retrospective cohort	833	Single institution	Cervical cancer patients	DFS, DMF, and OS: Histology, FIGO stage, para-aortic LNM, pelvic LNM, number of MLNs, and diameter of pelvic MLNs.
Yan et al. (2019) [41]	China	Retrospective cohort	485	Single institution	Early-stage cervical cancer patients	LVSI is an independent factor that affects OS and PFS.
Anfinan et al. (2020) [42]	Saudi Arabia	Retrospective cohort	190	Single institution	Cervical cancer patients	Survival was independently associated with grade II, grade III, number of regional organs involved, and recurrence.
Fang et al. (2020) [43]	China	Retrospective cohort	248	Multi-institutional	Early-stage cervical cancer patients	Higher rad-scores (radiomic score) were significantly associated with worse DFS.
Hou et al. (2020) [44]	Taiwan	Retrospective cohort	123	Single institution	Cervical cancer patients	CSS: The performance status, pathology with SCC, FIGO stage, and ICRT application were prognostic factors.
Kim et al. (2020) [45]	South Korea	Retrospective cohort	47	Single institution	Patients with early-stage high-grade neuroendocrine cervical carcinoma	DFS and OS: LNM and resection margin involvement were significant risk factors.
Lee et al. (2020) [46]	South Korea	Retrospective cohort	270	Multi-institutional	Locally advanced cervical cancer patients	DFS: Age, FIGO stage, tumor size, serum SCC antigen levels, pSUVmax, and nSUVmax. OS: HPV status, age, FIGO stage, tumor size, serum SCC antigen levels, pSUVmax, and nSUVmax.
Paik et al. (2020) [47]	South Korea	Retrospective cohort	1,441	Multi-institutional	Early-stage cervical cancer patients	DFS: Para-aortic LNM, histology, LVSI, depth of invasion (DI), pelvic LNM, and pretreatment serum hemoglobin (Hb). OS: Para-aortic LNM, histology, LVSI, depth of invasion (DI), cancer stage, and pretreatment serum hemoglobin (Hb).
Xie et al. (2020) [48]	China	Retrospective cohort	647	Single institution	Early-stage cervical cancer patients	DFS: Pelvic LNM, parametrial involvement, and cervical invasion greater than two-thirds depth. OS: Pelvic LNM and parametrial involvement.
Feng et al. (2021) [49]	China	Retrospective cohort	695	Single institution	Patients with primary cervical cancer	DFS and OS: Prior HBV infection was an independent prognosticator.
Guo et al. (2021) [50]	China	Retrospective cohort	5,112	Multi-institutional	Cervical cancer patients	FIGO stage, adjuvant therapy, parametrial involvement, histology, tumor size, DSI, and LN metastasis were prognosticators.
Kawamura et al. (2021) [51]	Japan	Retrospective cohort	65	Multi-institutional	Patients with Small cell carcinoma of the cervix (SCCC).	FIGO IVB at diagnosis was a key prognostic factor. Among patients who received chemotherapy, the SCCC regimen was associated with better 5-year OS.
Phung et al. (2021) [52]	Vietnam	Retrospective cohort	83	Single institution	Patients with FIGO 2018 stage III cervical cancer	DFS: The invasion of the lower third of the vagina, para-aortic LNM, and a pelvic LN short axis diameter of less than 15 mm were found to be unfavorable prognostic factors.
Shou et al. (2021) [53]	China	Retrospective cohort	116	Single institution	Patients with FIGO 2018 stage IIB-IVB cervical cancer	SUVmax (maximum standardized 18 F-fluorodeoxyglucose uptake value) of the cervix and FIGO 2018 stage were independent indicators of poor outcomes in squamous cervical cancer.
Tharavichitkul et al. (2022) [54]	Thailand	Retrospective cohort	295	Single institution	Cervical cancer patients	OS: non-SCC pathology, advanced stage, presented LN and longer OTT.
Chen et al. (2023) [55]	China	Retrospective cohort	251	Multi-institutional	Early-stage cervical cancer patients	Prognostic factors: FIGO stage, histological subtypes, LVSI, risk score.

Distribution of the Studies Across Asia

The distribution of publications on cervical cancer prognostic factors varies notably across regions. The majority of publications were from East Asia, Southeast Asia, and West Asia. China [22,24,27,28,32, 33,35,37,40,41,43,48-50,53,55] led with the highest number of publications (16), followed by Japan [14,17,19,23,26,36,38,51] and South Korea [12,13,21,29,34,45-47], each contributing eight papers. Taiwan [16,18,20,25,30,44] and Thailand [15,31,54] had six and three papers, respectively. Vietnam [52], Indonesia [39], and Saudi Arabia [42] each had one paper.

Prognostic Factors of Cervical Cancer in Asia

The most common socio-demographic factor associated with cervical cancer prognosis is age, as observed in five studies [12,15,16,30,46]. Regarding health-related factors, body mass index (BMI) has also been identified as an independent prognostic factor (P < 0.05) in a study conducted at the Hospital of Wenzhou Medical University, China, which included 795 patients with early-stage cervical cancer, FIGO grade IA1-IIA [32]. Medical comorbidities, such as anemia and diabetes mellitus, have also been associated with cervical cancer prognosis [20,35]. Anemia before radiotherapy was found to be an independent factor affecting overall survival (OS) in a multivariate analysis of 98 patients with cervical carcinoma (P = 0.008) [35]. Additionally, diabetes mellitus was identified as an independent unfavorable prognostic indicator for both OS (adjusted HR: 1.55) and cancer-specific survival (CSS) (adjusted HR: 1.46) after adjusting for comorbidities and clinicopathologic factors [20].

**Figure 2:**
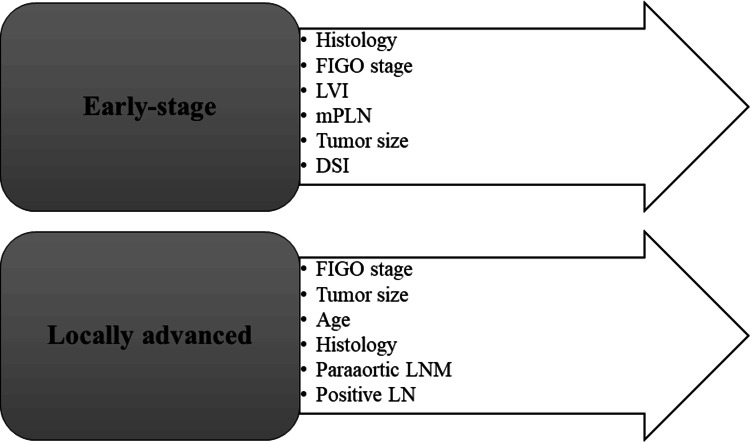
Prevalent prognostic factors in early-stage and locally advanced cervical cancer FIGO: International Federation of Gynecology and Obstetrics; LVI: lymphovascular invasion; DSI: deep stromal invasion; LNM: lymph node metastasis; mPLN: metastatic pelvic LN Image credit: Author Syed S Abrar

Among these, 16 studies focused on early-stage cervical cancer patients [12,15,20,24,25,28,31,34, 37,38,41,43,45,47,48,55], four on locally advanced patients [13,14,18,46], and one specifically on late-stage cancer patients [26]. The most common prognostic factors in early-stage and locally advanced cervical cancer patients are depicted in Figure 2.

Clinical factors: Cancer stage was consistently associated with cervical cancer prognosis across 16 studies [15,16,22,25,26,30,33,36,39,40,44,46,47,50,53-55]. The impact of histology as a prognostic factor was evaluated in several studies, with non-squamous cell carcinoma histology being particularly associated with a poor prognosis [13,18,19,21,29,30,36,37,40,54,55]. Adenocarcinoma, specifically, was identified as an independent poor prognostic factor in multiple studies [18,21,29,37]. In a Korean study involving 1,113 patients, adenocarcinoma was significantly associated with worse disease-free survival (DFS) (P = 0.0034) and OS (P = 0.0001) in multivariate analysis [29].

Tumor size, including the tumor diameter, was identified as an independent factor for cervical cancer prognosis in nine studies [13,14,17,19,30,32,35,46,50]. A Japanese study examining the medical histories of individuals with cervical cancer at stages IB2 to IIB found that a tumor size exceeding 60 mm was associated with a poorer overall prognosis [14]. Another study, involving 667 individuals in the early stages of cervical cancer, identified tumor diameter as a significant indicator of both OS and DFS [17].

Other significant clinical prognostic factors included lymphovascular invasion (LVI), lymph node (LN) metastasis, deep stromal invasion (DSI), the number of positive LNs, the number of metastatic LNs, parametrial involvement, and invasion [12,13,14,15,18,25,27,28,31,33,34,37,40,42,45,47,48,50,52,55]. Both pelvic and para-aortic LN metastasis were reported as prognostic indicators [13,27,34,37,40,47,52]. The status of HPV was also related to a worse prognosis in studies conducted in Korea and Japan [23,46]. A three-institution study evaluating 270 patients found that negative HPV status was linked to worse OS [46].

The study also identified serum squamous cell carcinoma (SCC) antigen levels, primary tumor maximum standardized uptake value (pSUVmax), and nodal maximum standardized uptake (nSUVmax) as independent indicators for DFS [46]. Pretreatment hemoglobin (Hb) levels were found to be adversely associated with prognosis in two studies [18,19,47]. An analysis of 1,441 early-stage patients reported that pretreatment Hb levels were a significant predictor for DFS [27]. A retrospective study of 178 women with cervical cancer at FIGO stages IB2-IVA observed that a pretreatment hemoglobin level of ≥11 g/dl vs. <11 g/dl was a significant indicator for predicting PFS (P = 0.01) [19].

Treatment-related factors: The reviewed studies did not fully explore treatment-related factors and their association with prognosis. However, adjuvant therapy was identified as a prognostic factor for OS (P = 0.041) in a multi-center study of 5,112 women with cervical cancer [50]. The effects of chemotherapy (P < 0.0001), treatment with extended-field radiation therapy (EFRT) (P = 0.003), and treatment intensity (P < 0.001) were also significant prognostic indicators [19,36,51]. Longer overall treatment time (OTT) was an adverse factor for prognosis in two separate studies [30,54]. Another study reported that treatment without brachytherapy (OS: HR 1.84 and CSS: HR 1.93, P < 0.001) and chemotherapy (OS: HR 2.04 and CSS: HR 1.46, P < 0.001) were also significant factors for OS and CSS [30].

Uncommon factors: Several lesser-reported prognostic indicators in Asian cervical cancer patients include elevated carboxypeptidase E (CPE) expression, surgery at high-volume centers, pre-treatment magnetic resonance imaging (MRI)-based radiomic scores, the number of regional organs involved, and prior HBV infection [24,38,42,43,49].

Neuroendocrine carcinoma of the cervix: In the U.S., neuroendocrine carcinoma of the cervix is a rare malignancy, comprising about 2% of all cervical cancers, with a mean annual incidence of 0.06 per 100,000 women. The mean age at diagnosis is around 45 years, and the prognosis is significantly worse compared to more common types, such as squamous cell carcinoma or adenocarcinoma [56]. Prognostic factors associated with neuroendocrine carcinoma of the cervix and small cell carcinoma of the cervix in Asian patients included age at diagnosis, FIGO stage, non-SCC histology, LN metastasis, tumor size, infiltration depth, and resection margin involvement [15,22,33,45,51].

**Figure 3:**
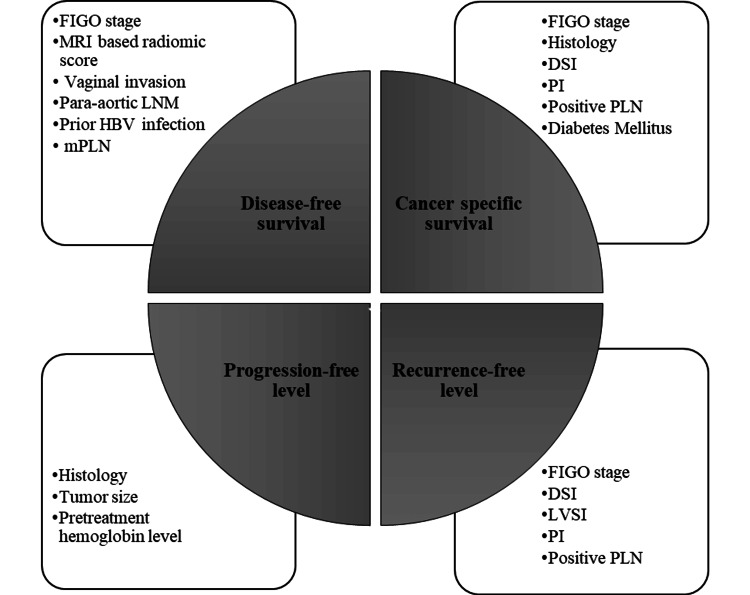
Prognostic factors related to DFS, RFS, PFS, and CSS DFS: disease-free survival; RFS: recurrence-free survival; PFS: progression-free survival; CSS: cancer-specific survival; FIGO: International Federation of Gynecology and Obstetrics; LVI: lymphovascular invasion; DSI: deep stromal invasion; LNM: lymph node metastasis; PI: parametrial involvement; mPLN: metastatic pelvic LN Image credit: Author Syed S Abrar

Among the 44 articles included in the review, most reported prognostic factors related to overall survival, while a few documented factors associated with disease-free survival (DFS) [17,18,22,23,29,34,40,43,45-49,52], recurrence-free survival (RFS) [25,31,37], progression-free survival (PFS) [19,41], and cancer-specific survival (CSS) [20,25,30,44]. Figure 3 illustrates the common factors observed to be associated with DFS, RFS, PFS, and CSS. 

Discussion

The identification and analysis of prognostic factors for cervical cancer in Asian populations revealed several key insights, emphasizing regional variations and the importance of various clinical, socio-demographic, and treatment-related factors. The geographical distribution of studies shows a concentration of research in East Asia, with China, Japan, and South Korea contributing the most publications. This underscores the scarcity of research on cervical cancer prognostic factors in West, Central, and South Asian countries. This regional focus suggests that the findings may be particularly relevant to East Asian populations, although the results could have broader implications across Asia. Most of the countries conducting research are either upper-middle- or high-income, including Saudi Arabia and Taiwan, highlighting the lack of research from low- and lower-middle-income countries.

The prognostic factors were consistent with those reported in non-Asian populations. The studies included in this review underscore the importance of socio-demographic factors, including age and health-related factors like BMI. Analysis of 46,350 women with cervical cancer revealed that older women show poor survival outcomes across all types of histology and regardless of disease stage, thus confirming the significance of age as a prognostic factor [57]. 

The significance of lymphovascular invasion, lymph node metastasis, and deep stromal invasion underscores their roles as clinical factors in predicting cervical cancer outcomes. This aligns with findings from recent global studies that highlight these factors' importance in prognosis [58]. Additionally, studies have shown that tumor characteristics like size and histology significantly influence outcomes. Notably, tumor size, particularly diameters exceeding 60 mm, consistently emerges as a critical predictor of poorer overall survival (OS) and disease-free survival (DFS). An analysis of 18,649 cervical cancer patients from the SEER database revealed that smaller tumor sizes were associated with enhanced cancer-specific survival (CSS), with a significant p-value of 0.0003 based on Kaplan-Meier estimates. Conversely, larger tumor sizes correlated with significantly poorer hazard ratios in both univariate and multivariate modeling. Specifically, the adjusted hazard ratios for tumor sizes of 40-59 mm and greater than 60 mm were 1.80 (CI 1.56-2.08, P < 0.0001) and 2.85 (CI 2.45-3.31, P < 0.0001), respectively [59]. Furthermore, non-squamous cell histology, especially adenocarcinoma, was identified as an independent poor prognostic factor, reaffirming the aggressive nature of these tumor types. This was in accordance with previous studies that have reported adenocarcinoma to have a poorer prognosis than squamous cell cervical cancer [60,61].

Interestingly, treatment-related factors were less explored, yet the available data suggest that adjuvant therapy, chemotherapy, and treatment intensity significantly impact survival outcomes. The adverse effect of longer OTT on prognosis, as well as the importance of brachytherapy and chemotherapy, highlights areas where treatment protocols could be optimized. The adverse prognostic effect of prolonged treatment time has been previously confirmed in several studies [62-64].

Finally, the review included studies on rare neuroendocrine carcinomas of the cervix, which, although constituting a small percentage of cervical cancers, present with a particularly poor prognosis as outlined in a recent review [65]. The identified prognostic factors for this subtype, such as FIGO stage, lymph node metastasis, and tumor size, mirror those found in more common types, albeit with greater severity of outcomes.

## Conclusions

In summary, this scoping review reported the various prognostic factors associated with cervical cancer prognosis in Asian populations. It underscores the critical need for research focused on low- and middle-income countries across Asia, where cervical cancer cases are on the rise. Key factors such as tumor characteristics, socio-demographic influences, and comorbidities emphasize the importance of a holistic approach to patient care. The prognostic factors observed in Asian populations are largely consistent with global findings, reaffirming their universal relevance. The review also points to the significant role of treatment-related factors in survival outcomes and suggests potential areas for optimization in treatment protocols. Furthermore, it draws attention to the scarcity of research on cervical cancer prognostic factors in West, Central, and South Asia, advocating for future studies to fill these gaps and enhance understanding across the region.
